# Giant hemorrhagic hamartoma of the Brunner’s of duodenum: a case report

**DOI:** 10.1093/jscr/rjag463

**Published:** 2026-06-13

**Authors:** Rodrigue Namèkinsba Doamba, Michael Osseis, Chetana Lim, Souleymane Ouattara, Adama Sanou, Daniel Azoulay, Chady Salloum

**Affiliations:** Department of General Surgery, Tengandogo University Hospital, 11 BP 104 CMS, Ouagadougou 01, Burkina Faso; Department of Visceral and Digestive Surgery, Saint Joseph University Hospital, Damascus Road, PO Box 17-5208, Mar Mikhael, Beirut 1104 2020, Lebanon; Department of Digestive, Hepatobiliary, and Pancreatic Surgery and Liver Transplantation, La Pitié-Salpêtrière Hospital, 47-83 Boulevard de l'Hôpital, 75013 Paris, France; Department of General Surgery, Tengandogo University Hospital, 11 BP 104 CMS, Ouagadougou 01, Burkina Faso; Department of General Surgery, Tengandogo University Hospital, 11 BP 104 CMS, Ouagadougou 01, Burkina Faso; Hepatobiliary Center, Paul Brousse Hospital, Villejuif, 12 Avenue Paul Vaillant Couturier, 94800 Villejuif, France; Hepatobiliary Center, Paul Brousse Hospital, Villejuif, 12 Avenue Paul Vaillant Couturier, 94800 Villejuif, France

**Keywords:** Brunner’s gland hamartoma, duodenum, treatment

## Abstract

A 56-year-old woman presented with hematemesis and melena. She was hemodynamically stable, with hemoglobin at 5.2 g/dL. Upper gastrointestinal endoscopy after transfusion showed twisting of the second duodenum (D2). Computed tomography and magnetic resonance imaging revealed intussusception caused by a duodenal mass without ischemia. Endoscopic ultrasound was inconclusive, and tumor markers were normal. Exploratory laparotomy found no peritoneal carcinomatosis. Duodenotomy exposed a 7 cm pedunculated submucosal tumor in D 2. Cannulation of the cystic duct identified the papilla, and an Escat drain was inserted. The mass was resected using Endo-GIA. Postoperative recovery was uneventful; the drain was removed on Day 3, and the patient was discharged on Day 4. Histopathology confirmed a completely resected benign Brunner’s gland harmartoma. After four years, no recurrence was observed. Brunner’s gland hamartomas are benign tumors. Surgery is indicated when endoscopic treatment is not feasible.

## Introduction

Brunner’s gland hamartomas are polypoid lesions rarely found in the duodenum. They account for 5%–10% of benign duodenal tumors and typically occur in middle-aged patients, with no gender predominance [1, 2].

They are usually small and asymptomatic. However, when they enlarge, they may cause duodenal obstruction or gastrointestinal bleeding. Management may be either endoscopic or surgical [2]. We report a rare case of a large pedunculated Brunner’s gland hamartoma of the duodenum presenting with gastrointestinal bleeding, with the aim of discussing diagnostic approach, management, and postoperative outcomes.

## Case presentation

A 56-year-old woman presented to the emergency department with profuse hematemesis and melena for 24 hours. She had no significant past medical history.

On admission, she was hemodynamically stable, with a blood pressure of 112/68 mmHg, heart rate of 107 beats/min, and oxygen saturation of 100%. Laboratory tests revealed a hemoglobin level of 5.2 g/dL.

**Figure 1:**
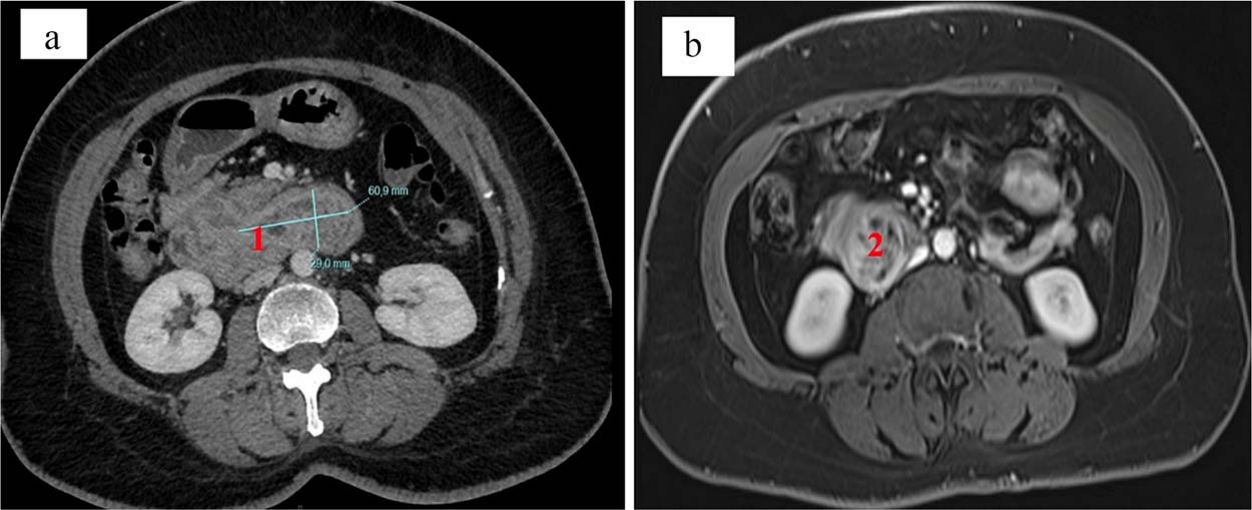
Appearance on imaging. Duodenal tumor on (a) CT scan and (b) MRI.

The patient received four units of packed red blood cells before undergoing upper gastrointestinal endoscopy, which revealed a twisted appearance of D2 without ulceration. Thoracoabdominal computed tomography (CT) scan showed intussusception involving a duodenal mass without signs of bowel ischemia. Magnetic resonance imaging (MRI) suggested volvulus of the second portion of the duodenum secondary to a mass (Fig. 1). Endoscopic ultrasound was performed twice but was inconclusive. Tumor markers were within normal limits (CA 19–9: 4.6 U/mL; CEA: 0.7 U/mL).

**Figure 2:**
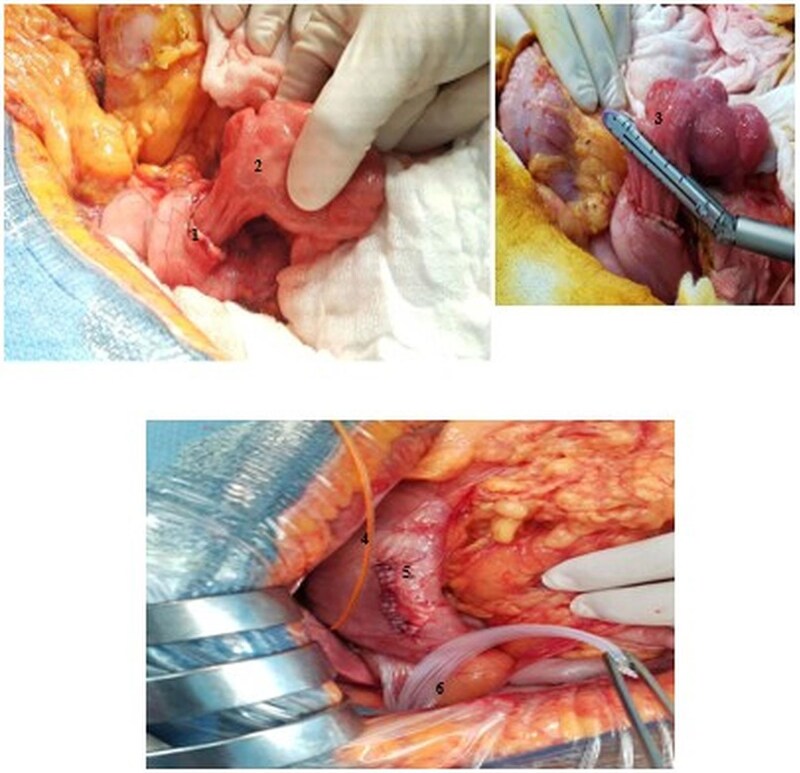
Intraoperative view. (1) Duodenotomy, (2) pedunculated tumor, (3) tumour resection by Endo-GIA stapling, (4) escat drain, (5) closure of the duodenotomy, (6) multitubular blade in front and behind duodenal suture.

The case was discussed in a multidisciplinary team meeting, and due to high suspicion of a duodenal tumor, exploratory laparotomy was performed. No peritoneal carcinomatosis or liver metastases were found. After Kocher maneuver and mobilization from D2 to D3, a longitudinal duodenotomy revealed a large pedunculated submucosal mass of 7 cm in D2. Cholecystectomy was performed, and the cystic duct was cannulated to identify the papilla using an Escat drain. The tumor was resected using two Endo-GIA staplers. Hemostasis was secured with PDS 4–0 sutures along the staple line. The duodenotomy was closed in two layers (mucosal and seromuscular), and a multitubular drain was placed anterior and posterior to the suture line (Fig. 2). The postoperative course was uneventful. The drain was removed on postoperative Day 3, and the patient was discharged on Day 4. Histological examination confirmed a Brunner’s gland hamartoma without malignancy and with complete resection. It was associated with mild, inactive antral gastritis and focal intestinal metaplasia without dysplasia. The patient remains recurrence-free four years after surgery.

## Discussion

Brunner’s gland hamartomas are rare benign duodenal tumors of unknown etiology. Factors such as *Helicobacter pylori* infection and chronic pancreatitis have been suggested in the literature [2].

The age of our patient falls within the commonly reported range (50–60 years). Rare cases have been described in younger patients aged 12–26 years [2, 3]. These lesions are usually small, with an average size of 2 cm, although giant forms up to 12 cm have been reported [4].

In our case, the tumor measured 7 cm. They are generally asymptomatic and often discovered incidentally. Symptoms, when present, are related to gastrointestinal complications.

The most common manifestations include dyspepsia, obstruction, diarrhea, gastroesophageal reflux, and occult or massive gastrointestinal bleeding [5]. Our patient presented with massive gastrointestinal bleeding. Diagnosis is often made during endoscopy performed for digestive symptoms or bleeding, and occasionally in emergency settings for obstruction. However, endoscopy may be limited by tumor size or duodenal distortion, as in our case. Additional imaging modalities such as endoscopic ultrasound, CT, and MRI are useful for lesion characterization and therapeutic planning [6, 7]. Most Brunner’s gland hamartomas are located in the duodenal bulb (57%) [8], whereas in our case the lesion was located in D2. Endoscopic resection is the treatment of choice, being less invasive and often not requiring hospitalization [8]. Surgical treatment remains indicated in cases of diagnostic uncertainty or when the lesion is not amenable to endoscopic removal. Although benign, cases with dysplastic changes have been reported, suggesting a potential—though rare—risk of malignant transformation [9, 10].

## Conclusion

Brunner’s gland hamartoma is a rare benign duodenal tumor. Its nonspecific presentation may delay diagnosis. It should be considered in cases of gastrointestinal bleeding. Imaging and endoscopy play key roles in diagnosis. Endoscopic treatment remains the first-line approach when technically feasible.
